# Residual stress measurements via neutron diffraction of additive manufactured stainless steel 17-4 PH

**DOI:** 10.1016/j.dib.2017.06.027

**Published:** 2017-06-16

**Authors:** Mohammad Masoomi, Nima Shamsaei, Robert A. Winholtz, Justin L. Milner, Thomas Gnäupel-Herold, Alaa Elwany, Mohamad Mahmoudi, Scott M. Thompson

**Affiliations:** a Laboratory for Fatigue & Additive Manufacturing Excellence (FAME), Department of Mechanical Engineering, Auburn University, Auburn, AL 36849, United States; b Department of Mechanical & Aerospace Engineering, University of Missouri, Columbia, MO 65211, United States; c Center for Neutron Research, National Institute of Standards and Technology, Gaithersburg, MD 20899, United States; d Department of Industrial and Systems Engineering, Texas A&M University, College Station, TX 77843, United States

**Keywords:** Additive manufacturing, Selective Laser Melting (SLM), Powder bed fusion, Residual stress, Heat treatment, microstructure

## Abstract

Neutron diffraction was employed to measure internal residual stresses at various locations along stainless steel (SS) 17-4 PH specimens additively manufactured via laser-powder bed fusion (L-PBF). Of these specimens, two were rods (diameter=8 mm, length=80 mm) built vertically upward and one a parallelepiped (8×80×9 mm^3^) built with its longest edge parallel to ground. One rod and the parallelepiped were left in their as-built condition, while the other rod was heat treated. Data presented provide insight into the microstructural characteristics of typical L-PBF SS 17-4 PH specimens and their dependence on build orientation and post-processing procedures such as heat treatment. Data have been deposited in the Data in Brief Dataverse repository (doi:10.7910/DVN/T41S3V).

**Specifications Table**

**Table:** 

Subject area	Mechanical Engineering
More specific subject area	Additive Manufacturing
Type of data	Tables; Graphs; Excel Worksheets
How data was acquired	Neutron diffraction, BT8 neutron diffractometer at National Institute of Standards and Technology (NIST) Center for Neutron Research (CNR)
Data format	Raw and analyzed
Experimental factors	Three stainless steel (SS) 17-4 PH specimens were fabricated from gas-atomized powder using laser powder bed fusion (L-PBF). Optimized process parameters were employed to generate two vertical rods and a similar-dimensioned, horizontal parallelepiped. Specimens were removed from the build plate using electrical discharge machining (EDM). Heat treatments (solution annealing and aging) were applied to one of the as-built, cylindrical specimens. The other two specimens remained in their as-built condition. Specimen surfaces were cleaned.
Experimental features	Residual stresses at specific locations along the radius and length of cylindrical rods and the x,y,z directions for the parallelepiped were measured using neutron diffraction. Effects of heat treatment and build direction on the residual stress distribution in L-PBF SS 17–4 PH may be determined using the presented tables and plots.
Data source location	NIST CNR, Gaithersburg, Maryland, USA
Data accessibility	Data have been deposited in the Data in Brief Dataverse repository (doi:10.7910/DVN/T41S3V).
	https://dataverse.harvard.edu/dataset.xhtml?persistentId=doi:10.7910/DVN/T41S3V

**Value of data**

• Residual stress can lead to premature fatigue failure and deformation of parts. Therefore, understanding and characterizing residual stress is important for ensuring part reliability. Data provided aid in characterizing residual stress distributions in specimens fabricated via laser powder bed fusion (L-PBF) and other directed energy, powder-based additive manufacturing (AM) methods.
• Data can be used to explain fatigue and deformation behavior of AM parts observed by others.
• Data demonstrate effects of heat treatment and building orientation on residual stress distributions in stainless steel (SS) 17-4 PH specimens made via L-PBF.
• Data provide a means to generate and validate numerical and/or analytical thermomechanical models for their prediction of residual stress in AM parts.
• Data can be used as an educational tool for learning how to calculate residual stresses given raw measurements obtained via neutron diffraction of metals.
• Data may be compared with residual stress measurements found via other techniques.

## 1 Data

The residual stress within heat treated and as-built (or, ‘as-is’) stainless steel (SS) 17-4 PH specimens fabricated via laser powder bed fusion (L-PBF) were measured using neutron diffraction at NIST׳s Center for Neutron Research (CNR). The presented data include measured lattice strains (i.e. d-spacings), stress-free lattice spacings (*d*_0_) and hoop/axial (or x-,y-,z-component) residual stress calculations. Uncertainties associated with residual stress measurements are estimated and also provided. All results are presented in the form of tables and plots in multiple Excel worksheets. Three specimens were analyzed and their corresponding measurements are grouped by tab color, i.e.: vertical as-is (i.e. as-built) rod (color code=red), vertical/heat-treated as-is rod (color code = blue), and the horizontal as-is parallelepiped (color code=yellow). Comment boxes are provided in the Excel sheets with instructions on how to replicate calculations using X-ray diffraction data analysis software. Data are supported with schematics that indicate the diffraction locations and manufacturing scan patterns.

## 2 Experimental design, materials and methods

A PHENIX PM-100 Selective Laser Melting (SLM) system equipped with a 50 W Nd:YAG laser was utilized for the L-PBF of specimens from gas-atomized, stainless steel (SS) 17-4 PH powder (Phenix Systems) feedstock. The powder feedstock possessed a size distribution of: 10 μm<D50<13.5 μm and D80<22 μm [1]. All specimens were built together on the same, non-heated substrate within an argon-purged environment. Two vertical rods and a horizontal parallelepiped were manufactured. The cylindrical specimens were approximately 8 mm in diameter and 80 mm in height. Each layer of the parallelepiped possessed dimensions of 8×80 mm^2^ and its total height was 9 mm. Process parameters (i.e. laser power, scanning speed, layer thickness, and hatching pitch) were optimized to obtain an acceptable level of final part density using a design of experiments methodology [1]. The final process parameters used, which are summarized in Table 1, included: laser power of 48 W, traverse speed of 300 mm/s, layer thickness of 30 μm, and hatch spacing of 50 μm.

**Table 1:** Parameters used for fabricating specimens.

Powder and substrate material	SS 17-4 PH
Powder description	Gas-atomized, air-dried
Powder size distribution	10 μm<D50<13.5 μm
Powder layer thickness	30 μm
Hatch spacing	50 μm
Laser spot diameter	70 μm
Laser power	48 W
Laser wavelength	1075 nm
Scan speed	300 mm/s
Shielding gas type	Argon
Shielding gas temperature	20 °C
Shielding gas flow rate	167 cm^3^/s
Substrate temperature	20 °C

**Fig. 1:**
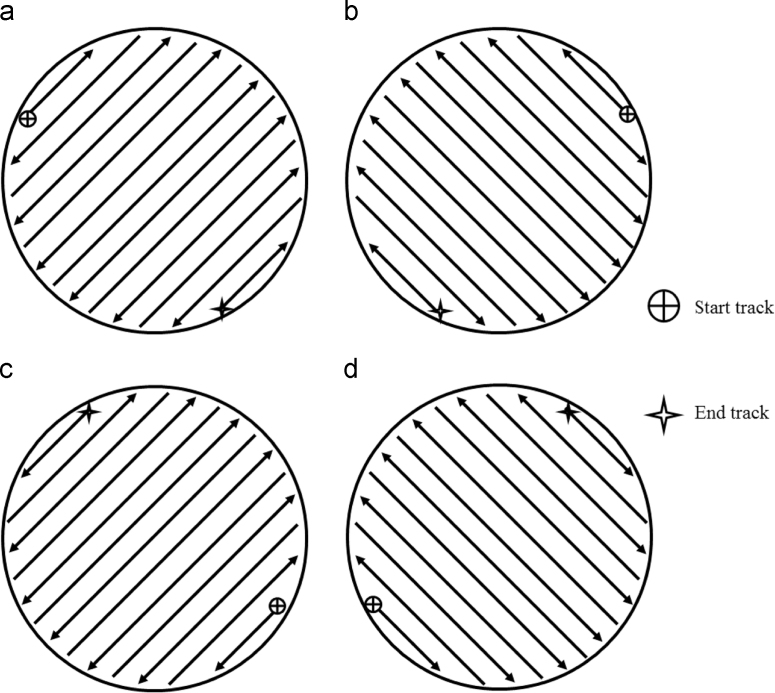
Scan strategy for vertical sample for the first through fourth layers (a)–(d), respectively. Successive layers are a repeat of these four in the same order.

**Fig. 2:**
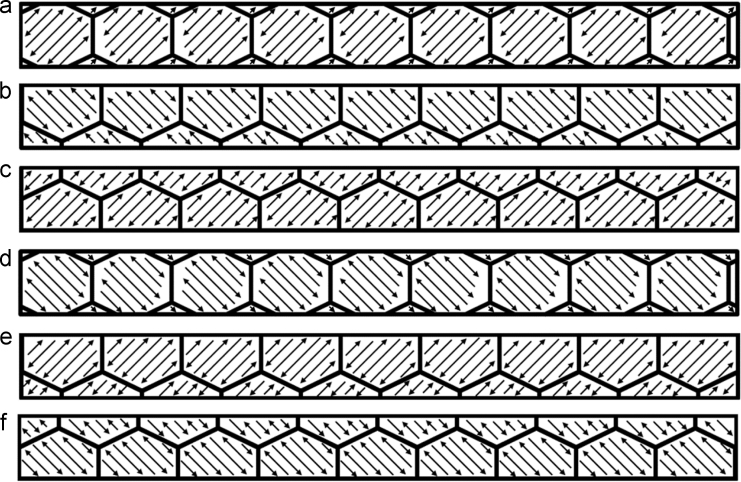
Scan strategy for horizontal sample layers one through six, (a)–(f), respectively. Successive layer are a repeat of these six in the same order.

Default scan strategies were used for fabricating each specimen. For the vertical rods, the laser started at the top left region of the first layer as shown in Fig. 1(a). The laser then moved back and forth in a hatching pattern until the layer was complete. For the second layer, the same hatch pattern was repeated; only it was rotated 90° clockwise, as shown in Fig. 1(b). The scan patterns for the third and fourth layers were similar, however, they were rotated 180° and 270° clockwise relative to the first scanning directions, as shown Fig. 1(c) and (d). This scan strategy was repeated after completion of the fourth layer until the end of the build. For the parallelepiped, the scan strategy consisted of building several, equal-sized hexagonal regions (~5 mm in length) in a random order. The hexagonal scan strategy varied with each layer as shown in Fig. 2. The layer-wise scanning strategy outlined in Fig. 2 was repeated after completion of every 6th layer.

Electrical discharge machining (EDM) was employed to remove specimens from the substrate. Samples were not thermally stress relieved prior to their removal. In order to investigate the effect of heat treatment, one of the as-built rods underwent solution annealing (Condition A) followed by peak-aging (Condition H900) [2]. The final microstructures consisted of a mixture of ferrite and austenite. All specimen surfaces were cleaned of any loose powder.

Lattice strains (i.e. d-spacings) were measured along orthogonal directions at pre-selected 1×1×1 mm^3^ regions (i.e. gage volumes) of the specimens using the BT8 neutron diffractometer at NIST׳s CNR. Employed neutrons originated from a continuous, cold source. The BT8 residual stress diffractometer possessed three monochromators and a rotating drum (for inspecting multiple specimen orientations) to allow for wavelength variation between 0.8 and 3.2 Å and measurement of d-spacings between 0.56 and 2.26 Å. The neutron beam wavelength was set to 1.637 Å. An Ordela 1150 position sensitive neutron detector with an angular opening of approximately 8° was employed. The adopted measurement method used several pole figures for each phase, which in this case was austenite and ferrite, to obtain an orientation average of the *hkl*-dependent peak intensity [3]. The techniques used herein are explained in detail elsewhere [4], [5].

Residual stresses were calculated using Bragg׳s law with many of the coefficients provided in Columns J-W in the Excel worksheets. Due to the weak attenuation of neutrons, their penetration depth is higher than X-rays [4]. Diffraction from the {311} planes at 2*θ*=95.89° and {211} planes at 2*θ*=88.77° were used for analyzing the austenite and ferrite phases, respectively. It took approximately 1 hour to collect neutron diffraction data per diffraction peak. Due to time constraints, it was not possible to perform the elastic constants measurements. Instead, the isotropic diffraction elastic constants were calculated using the Kröner model as described in [6]. Note that each gage volume consists of approximately 33 layers, thus residual stress measurements are spatially averaged.

**Fig. 3:**
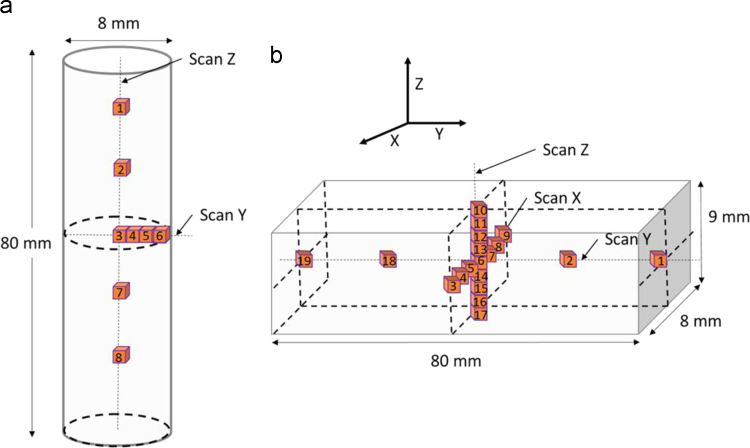
Measurement locations for (a) as-built and heat-treated cylindrical specimens and (b) as-built horizontal parallelepiped specimen.

The stress-free lattice spacing, *d*_0_, was calculated for each sample by utilizing near-surface measurements where the stress component normal to the surface can be presumed to be zero. In this case, radial stresses for cylindrical samples were presumed to be zero near the surface. This was done for each phase, and the weighted average was calculated. For the parallelepiped, *d*_0_ was estimated from measurements with locations close to surfaces in which either *σ*_xx_=0 or *σ*_zz_=0 was applicable, Fig. 3(b). Four different estimates for *d*_0_ were obtained, and the average was taken, thus obtaining a single *d*_0_ for each phase. This is a common method for circumventing the *d*_0_ problem [7]. The *d*_0_ calculations for the parallelepiped have a dedicated tab in the Excel file: “S3 *d*_0_”. The presence of a third phase due to precipitation hardening was not accounted for and therefore presents an unresolved uncertainty.

Measurement locations are presented in Fig. 3. Gage volumes were distributed along the y (radial) and z (axial) axes for cylindrical samples to find residual stress trends in these directions. As shown in Fig. 3(a), four measurement locations, #3, #4, #5 and #6, were distributed along the radial direction and five measurement locations were distributed along the axial direction, #1, #2, #3, #7 and #8. For the parallelepiped, 7, 5 and 9 measurement locations were distributed along the x, y and z directions, respectively. The gage volumes were evenly spaced in each direction. In Table 2, the hoop and axial stresses along with their uncertainties for the as-built rod are presented. In Table 3, the residual stress for these same points are presented for the heat-treated rod. Finally, the Cartesian component residual stresses for the as-is, horizontal parallelepiped at the aforementioned measurement locations are presented in Table 4.

**Table 2:** Residual stress and its uncertainty for vertical as-is sample.

Point #	Average hoop (MPa)	Uncertainty (MPa)	Average axial (MPa)	Uncertainty (MPa)
1	37.8	15.2	−52.4	14.4
2	−23.3	16.0	−35.3	15.9
3	35.2	13.8	−14.1	13.7
4	−1.8	13.0	−16.7	13.3
5	−3.1	12.9	−26.8	11.3
6	52.6	12.5	59.8	13.4
7	16.0	13.1	−23.4	12.8
8	7.3	11.7	−84.7	11.8

**Table 3:** Residual stress and its uncertainty for vertical heat-treated sample.

Point #	Average hoop (MPa)	Uncertainty (MPa)	Average axial (MPa)	Uncertainty (MPa)
1	−38.4	18.8	−33.6	17.0
2	25.9	17.4	−5.8	16.4
3	−27.4	18.8	8.4	18.4
4	−9.7	18.1	4.4	16.5
5	−10.5	16.2	−2.4	13.6
6	24.2	14.9	39.7	12.5
7	38.5	19.3	8.6	18.5
8	−148.8	18.1	−22.0	17.9

**Table 4:** Residual stress and its uncertainty for horizontal as-is sample.

Point #	*σ*_xx_ (MPa)	Uncertainty (MPa)	*σ*_yy_ (MPa)	Uncertainty (MPa)	*σ*_zz_ (MPa)	Uncertainty (MPa)
1	−34.8	23.3	−78.3	20.0	−57.7	20.9
2	−53.1	24.8	−27.5	20.4	−112.5	22.7
3	16.6	27.6	58.9	21.9	124.4	20.8
4	−21.6	26.9	1.0	21.5	−27.2	21.2
5	44.1	26.0	60.2	20.8	−7.5	21.8
6	−9.8	17.1	−11.1	14.6	−61.2	16.3
7	−1.1	20.6	−5.4	20.3	−67.2	20.8
8	−11.4	17.6	0.8	18.6	−20.4	19.2
9	−4.6	17.5	2.7	20.1	112.7	18.2
10	153.3	25.2	104.3	25.5	−82.0	24.1
11	53.3	19.5	60.9	18.6	−10.3	16.5
12	34.7	20.3	49.7	18.8	−3.9	18.2
13	−33.2	25.4	−9.7	20.5	−64.0	23.1
14	−50.5	25.3	−21.3	21.8	−71.5	28.0
15	−26.8	21.8	16.9	20.5	−48.0	25.8
16	70.3	21.1	71.9	20.0	16.3	25.7
17	223.6	22.2	145.5	23.1	50.4	26.6
18	−49.8	24.6	−30.3	21.1	−78.5	25.7
19	1.3	24.4	−30.5	20.7	−41.1	25.2

The results in the spreadsheet can be reproduced by downloading PeakFit (PF) at https://www.ncnr.nist.gov/instruments/bt8/PF.zip and pasting the spreadsheet contents into the stress calculation worksheet. When doing this, ׳D0׳ should be a fixed parameter, all other stresses should be “free” or unchecked. There are comment boxes in the Excel sheet providing instructions.

## 3 Disclaimer

An author of this article is currently serving on the editorial board of Data in Brief. Accordingly, the editorial and peer review process for this article was not handled by this author. Furthermore, all authors of this article do not have access to any confidential information related to its peer-review process.
