# Supplementary material for: Residual stress measurements via neutron diffraction of additive manufactured stainless steel 17-4 PH
Source: Data Brief. 2017 Jun 16;13:408–14. doi: 10.1016/j.dib.2017.06.027 (PMC5480826; doi:10.1016/j.dib.2017.06.027)
Supplement: Supplementary file 1 — Supplementary material [file mmc1.docx]

There are no perceived or actual conflict of interests.
